# Access to primaquine in the last mile: challenges at the service delivery points in pre-elimination era, Myanmar

**DOI:** 10.1186/s41182-018-0115-8

**Published:** 2018-09-18

**Authors:** Kay Thwe Han, Khin Thet Wai, Tin Oo, Aung Thi, Zayar Han, Daw Kyin Hla Aye, Aung Ye Naung Win, Jetsumon Prachumsri

**Affiliations:** 1 Parasitology Research Division, Department of Medical Research (DMR), No. 5 Ziwaka Road, Yangon, 11191 Myanmar; 2 DMR, Yangon, Myanmar; 3 National Malaria Control Program, Department of Public Health (DoPH), Yangon, Myanmar; 4 Parasitology Research Division, DMR, Yangon, Myanmar; 5 Epidemiology Research Division, DMR, Yangon, Myanmar; 6 Mahidol Vivax Research Unit, Faculty of Tropical Medicine, Mahidol University, Nakhon Pathom, Thailand

**Keywords:** Primaquine, Sustained access, Service delivery points, Antimalarial treatment guidelines, Pre-elimination, Myanmar

## Abstract

**Background:**

Alongside monitoring of the disease burden, the successful move towards malaria elimination relies on the readiness of the health care delivery system. However, there is a lack of evidence in the gap of existing National Guidelines and access to low dose primaquine in real practice under varying degrees of antimalarial resistance in the pre-elimination phase in Myanmar. Therefore, this study addressed the essential information from the service delivery points (SDPs) of public and private sectors on the availability and the use of primaquine in both supply and demand side. Concomitantly, the study aimed to underscore challenges in health system infrastructure to promote the sustained flow in rolling out primaquine in line with National Guidelines for malaria elimination.

**Methods:**

A cross-sectional study conducted from September 2017 to February 2018 included six townships of three states/regions. The team used an observation checklist for documenting primaquine supplies at SDPs. Semi-structured interviews, key informant, and in-depth interviews focused both public and private sectors including staff from the Vector-Borne Diseases Control (VBDC) teams in each state/region and rural health centers (*n* = 25), those from the non-governmental organizations (NGOs), general practitioners and drug sellers (*n* = 11), and recently infected malaria patients (*n* = 11). Triangulation of quantitative and qualitative data provided meaningful interpretations.

**Results:**

Public sector staff reported an adequate stock of primaquine, but it was unavailable at the general practitioners’ clinics without any connection to NGOs and also at the unlicensed drug shops. Health care providers of the public sector experienced challenges in poor compliance of malaria patients to primaquine treatment in conjunction with an artemisinin-based combination therapy, loss-to-follow-ups especially in conflict areas, and delays in timely substitution of new batches of primaquine. Respondents from the private sector demanded for the refresher training course on updated antimalarial treatment guidelines.

**Conclusion:**

Monitoring compliance and safety of primaquine treatment was found as a barrier especially among mobile migrant workers and those who were in conflict areas. An alternative strategy by the NMCP could enable to prevent the underutilization of primaquine in vivax malaria to reach the malaria elimination targets.

## Background

An access to a WHO-recommended low-dose primaquine for gametocytocidal action is critical in global elimination settings as one of the malaria transmission-blocking tools [1]. The National Malaria Control Program (NMCP) in Myanmar has launched the National Malaria Elimination Plan (2016–2030) for accelerating the progress towards zero indigenous cases. Above all, due to the considerable decrease in *Plasmodium falciparum* malaria, *Plasmodium vivax* is emerging and dominating in some endemic areas. However, malaria still prevails in hard-to-reach areas with an emerging evidence of drug resistance [2–4]. The National Malaria Treatment Guidelines indicate the use of artemisinin combination therapy (ACT) in conjunction with a gametocytocidal dose of primaquine of 0.25 mg/kg for both falciparum (single dose) and vivax malaria (8 weeks regimen) [5]. Alongside monitoring of the disease burden, the successful move towards malaria elimination relies on the readiness of the health care delivery system in conformity to other endemic regions [6–8]. However, there is a lack of evidence in the gap of existing National Guidelines and access to low dose primaquine in real practice under varying degrees of antimalarial resistance in the pre-elimination phase. Therefore, this study addressed the essential information from the service delivery points (SDPs) of public and private sectors on the availability and the use of primaquine in both supply and demand side. Concomitantly, the study aimed to underscore challenges in health system infrastructure to promote the sustained flow in rolling out primaquine in line with the National Guidelines for malaria elimination.

## Methods

A cross-sectional study was conducted from September 2017 to February 2018 in six study sites of three states/regions designated for malaria elimination by 2030. The study population encompassed the public sector health staff of the NMCP and at the SDPs in rural areas, those from the private sector viz., staff of non-governmental organizations (NGO), general practitioners (GP), and drug sellers, and recently infected malaria patients.

### Sampling and sample size

In each study site, the research team purposively selected two out of six to eight rural health centers (RHCs) with a higher number of reported malaria cases compared to others. At least 80% of health staff (*n* = 36) who were responsible to diagnose and treat malaria-infected patients, three informants from state/regional VBDC teams, one senior program official from the NGO, and ten respondents (seven GP and three drug sellers) were purposively selected for key-informant interviews (KII) and in-depth interviews (IDI). In addition, the research team used convenience sampling to recruit recently infected malaria patients (*n* = 11).

### Data collection methods

The research team used the *observation checklist* to record previous access to primaquine, details of drug supply, and the number of malaria cases tested and treated in each SDP at different levels along the supply chain (rural, township, and state/region). Then, trained interviewers conducted the *semi-structured interviews* of health care providers from the public sector (*n* = 25) as well as from the private sector (*n* = 11) by using the pre-tested and modified questionnaire to uncover their knowledge and experiences in prescribing primaquine, adherence to National Treatment Guidelines and challenges encountered. Pre-tested guidelines for KII and IDIs covered similar issues as semi-structured interviews in the private place. In addition, trained interviewers assessed the use and compliance to primaquine among recently infected malaria patients (*n* = 12), and anonymity and confidentiality issues were strictly observed.

### Data management

Quantitative data entry and analysis were carried out by SPSS version 23.0. After thematic analysis of qualitative data, triangulation of quantitative and qualitative data was done for meaningful interpretations.

## Results

### Access to primaquine

In the public sector, primaquine was available at the respective health facilities without any expired stock according to the clinic registers. In contrast, in the private sector, only five out of eight GPs’ clinics and the SDPs of two international NGOs had primaquine. Concerning the supply chain at the public sector, all SDPs had received primaquine from the state/regional VBDC teams. The nature of the supply chain was the “pull system,” a need-based system to fill up antimalarials either monthly or quarterly. At these SDPs, they did not report any stock-outs of primaquine within last 3 months. At the private sector, GP clinics supported by private not-for-profit NGOs were fully equipped with primaquine. However, other GP clinics without any NGO support purchased antimalarials from the retail market except for primaquine. The persons responsible for logistics management of two INGOs did not report any primaquine stock-outs in SDPs. Besides, they had a system to regular exchange of nearly expired batches with new batches of primaquine. Primaquine was not available at any of the drug shops surveyed. In some uncomplicated falciparum malaria and confirmed vivax malaria cases, primaquine use was not in line with treatment guidelines among providers in the private sector (Table 1).

**Table 1:** Reported antimalarial treatment practices of health care providers in six study sites of three states/regions, Myanmar (*n* = 32)

Health care	Treatment	Study region A	Study region B	Study region C	Total
Confirmed uncomplicated falciparum malaria					
Public sector (*n* = 24)	ACT + PQ (correct dose and course)	6/6 (100%)	10/10 (100%)	8/8 (100%)	24/24 (100%)
	Artesunate 2 tds × 3 days	0	0	0	0
Private sector (*n* = 8)	ACT + PQ (correct dose and course)	1/2 (50%)	1/2 (50%)	3/4 (75%)	5/8 (65%)
Confirmed vivax malaria					
Public sector (*n* = 24)	CQ + PQ (correct dose and course)	8/8 (100%)	5/5 (100%)	11/11 (100%)	24/24 (100%)
	CQ only	0	0	0	0
Private sector (*n* = 8)	CQ + PQ (correct dose and course)	0/3	0/4	0/1	0/8
	CQ only	0	0	0	0

*ACT* artemisinin combination treatment, *PQ* primaquine, *CQ* chloroquine, *tds* three times a day

### Challenges in sustained use of primaquine

The long course of primaquine treatment for 8 weeks in vivax patients was the main reason of poor compliance reported by health staff from the public sector. Mostly, patients rarely returned after 2 weeks. Moreover, health staff felt that it was time-consuming and costly for them to travel from SDPs in rural areas to the Township Health Department for substitution of the nearly expired primaquine with new batches. Respondents further addressed migrant workers as the risk groups in loss-to-follow-ups and found as difficult to monitor their treatment compliance and outcome. They had confronted the similar issue in conflict areas. On the other hand, health care providers of the private sector stated challenges such as a lack of chance to attend the continuing medical education sessions or the refresher training course on updated antimalarial treatment guidelines. Besides, they were more or less not familiar to the ideal treatment of malaria due to the decreasing number of malaria cases and infrequent experience. Primaquine was not available at the retail market, and ACT was the only treatment provided for malaria cases at private clinics without NGO support.

Also, they were not able to monitor the patients’ compliance as they failed to turn-up following recovery from the recent malaria episode.

### Opinions to improve primaquine treatment

In the public sector, the respondents preferred the shorter course of primaquine regimen in malaria due to *Plasmodium vivax* for better compliance than at present. Those from the remote areas would like to opt for the provision of primaquine within the acceptable expiry date to prevent immediate stock-outs and to improve easy access. In the private sector, the availability of primaquine in the retail markets could improve an easy access to GPs so as to treat the patients in accordance with National Treatment Guidelines as required.

## Discussion

In Myanmar, in low malaria endemic sites targeted for elimination by 2030, vivax malaria reemerged compared to the lower prevalence of falciparum malaria [3]. This study clearly demonstrates the critical role of the updated National Treatment Guidelines to be available for service providers especially in the private sector apart from the sustained access to primaquine in pre-elimination phase. On the other hand, prevailing parasite species in the low endemic settings moving towards elimination influenced the treatment decisions for primaquine and lack of monitoring for compliance to full course in both sectors. In falciparum malaria, the use of SLD primaquine and the adherence to treatment guidelines and compliance were well monitored as reported by key informants in this study similar to African context [9]. In vivax malaria, the longer course and the higher dose of primaquine were needed for radical cure to clear liver stage parasites which was the main reason for non-compliance to full course in this study. Public not-for-profit service providers raised this issue during key informant interviews for their reluctance to use primaquine rather than the safety concerns about hemolysis in Glucose-6-phosphate dehydrogenase (G6PD) deficiency and other contraindications as expected which was consistent to other studies [10, 11]. If vivax malaria is to be eliminated, the use of radical curative treatment requires attention. Due to divergence in policy and practice, primaquine is underused [12]. To date, Thailand and Cambodia have adopted malaria elimination in their national strategic plans to achieve zero indigenous case status by 2024 and 2025, respectively. Both country programs plan to introduce or revise guidelines for safe, effective radical cure for vivax malaria with a routine G6PD testing [11, 12]. However, policy alone is not sufficient. Programs can only gain from the policy if they are capable for planning and implementation in hard-to-reach areas. Allowing for sufficient time and specific guidance is critical to prepare for training and other activities to promote the use of low-dose primaquine particularly in remote sites.

### Limitations of the study

The study period covered only the second peak of malaria transmission season in Myanmar. Very few malaria patients could join the interviews and were unable to capture their constraints in compliance to long course of primaquine in vivax malaria.

## Conclusion

In both public and private sectors, monitoring compliance and safety of primaquine treatment was a barrier especially among mobile migrant workers and those who were in conflict areas. Introducing an alternative strategy by the NMCP is a necessity to improve treatment options and to prevent the underutilization of Primaquine for gametocytocidal effect intended in vivax malaria to reach the malaria elimination targets.

## Abbreviations

ACT: Artemisinin combination therapy
GP: General practitioner
IDI: In-depth interviews
KII: Key informant interviews
NGO: Non-governmental organizations
NMCP: National Malaria Control Program
RHC: Rural health center
SDPs: Service delivery points
VBDC: Vector-Borne Diseases Control
