# Correction: Access to primaquine in the last mile: challenges at the service delivery points in pre-elimination era, Myanmar

**DOI:** 10.1186/s41182-023-00499-8

**Published:** 2023-02-03

**Authors:** Kay Thwe Han, Khin Thet Wai, Tin Oo, Aung Thi, Zayar Han, Daw Kyin Hla Aye, Aung Ye Naung Win, Jetsumon Sattabongkot

**Affiliations:** 1 Mahidol Vivax Research Unit, Faculty of Tropical Medicine, Mahidol University, Nakhon Pathom, Thailand; 2 Parasitology Research Division, Department of Medical Research (DMR), No. 5 Ziwaka Road, Yangon, 11191 Myanmar; 3 DMR, Yangon, Myanmar; 4 Department of Public Health (DoPH), National Malaria Control Program, Yangon, Myanmar; 5 Parasitology Research Division, DMR, Yangon, Myanmar; 6 Epidemiology Research Division, DMR, Yangon, Myanmar

**Correction: Tropical Medicine and Health (2018) 46:32** 10.1186/s41182-018-0115-8

Following publication of the original article [1], the authors reported that the author affiliations need to be updated.

The original author affiliations were:

^1^ Parasitology Research Division, Department of Medical Research (DMR), No. 5 Ziwaka Road, Yangon 11191, Myanmar.

^2^ DMR, Yangon, Myanmar.

^3^ National Malaria Control Program, Department of Public Health (DoPH), Yangon, Myanmar.

^4^ Parasitology Research Division, DMR, Yangon, Myanmar.

^5^ Epidemiology Research Division, DMR, Yangon, Myanmar.

^6^ Mahidol Vivax Research Unit, Faculty of Tropical Medicine, Mahidol University, Nakhon Pathom, Thailand.

The correct author affiliations have been updated in this Correction.

The original article [1] has been corrected.
